# Spirulina-coenzyme Q10 nanoemulsion regulating growth, antioxidant, immune capacity, histopathological alterations in Nile tilapia exposed to heat stress

**DOI:** 10.1038/s41598-026-36000-8

**Published:** 2026-02-06

**Authors:** Shaimaa A. A. Ahmed, Walaa EL-Houseiny, Mohamed ElHady, Mohamed M. Metwally, Sameh H. Ismail, Wessam El-Shahat

**Affiliations:** 1 Department of Aquatic Animal Medicine, Faculty of Veterinary Medicine, Zagazig University, Zagazig, 44511 Egypt; 2 Department of Pathology, Faculty of Veterinary Medicine, Zagazig University, Zagazig, 44511 Egypt; 3 Faculty of Nanotechnology for Postgraduate Studies, Sheikh Zayed Branch Campus, Cairo University, Sheikh Zayed City, Giza, 12588 Egypt

**Keywords:** Heat stress, Nanoemulsion, Nile tilapia, *Streptococcus agalactia*e, Biochemistry, Microbiology, Zoology

## Abstract

Variation in water temperature has a big impact on fish physiology, behavior, growth, and other biological processes. The current perspective aimed to assess the ameliorative effects of Spirulina-co-enzyme Q10 nanoemulsion (SCN) dietary inclusion on reared Nile tilapia (*Oreochromis niloticus*) health aspects under heat stress conditions (32 °C). A random design was used to establish 5 groups of Nile tilapia (*n* = 225), each consisting of 15 fish in triplicates. After being given a baseline diet, the 1st (C25) and 2nd (SCN0) groups were raised at 25 °C and 32 °C respectively and supplemented by a basal diet. Diets supplemented with a10, 20, and 40 mg/kg diet were given to groups 3 (SCN10), 4 (SCN20), and 5 (SCN40), respectively, which were raised at 32 °C for 60 days. At the termination of the 60-day session, growth metrics, and immunological biomarkers were assessed. Tissue specimens were also obtained for further assessment of the antioxidant status and histopathological alterations. A subsequent bacterial challenge with *Streptococcus agalactiae* was performed, where mortalities, clinical symptoms, and postmortem lesions were investigated. As per the results of the investigation, dietary incorporation with SCN40 followed by SCN20 significantly promoted growth metrics and immunological biomarkers, where the lowest records were noted in SCN0. Tissue antioxidants (SOD, GST, and CAT) showed notable improvement in SCN40, which also expressed the lowest MDA value compared to SCN0. Nonetheless, oxidative stress mediators and lipid peroxidation markers revealed a marked decline in SCN-fortified groups in a concentration-associated pattern compared with the SCN0 group. Histopathological evaluation of liver, intestine, and spleen tissues revealed a noticeable improvement and almost restoration of the normal tissue architecture in the SCN20 and SCN40 groups. Following the *Streptococcus agalactiae* challenge, Nile tilapia survivability was significantly higher in SCN40 (80%) than in SCN20 (74%). Taken together, SCN, especially at 20 and 40 mg levels, demonstrated its valuable impact on mitigating heat stress in the Nile tilapia.

## Introduction

Globally, millions of people depend on aquaculture, a quickly growing food production sector, to provide them with high-protein foods like fish. Prospective fish consumption is a significant worry due to the anticipated population rise and the high proportion of undernourished individuals^1^. Nile Tilapia production poses a valuable issue in the aquaculture sector, reaching 4407.2 thousand tons, or 9% of the total volume, which candidates them as the third main reared fish worldwide^2^. In Egypt, Nile tilapia is ranked as the most prevalent cultured food fish^3^. Tilapia farming has several benefits, including quick growth, high production, and high-quality meat, as well as social and economic advantages^4,5^. In addition to its omnivorous diet, which encourages the adoption of living or inert items, Nile tilapia’s growth is associated with its tolerance to broad variations in salinity, temperature, and dissolved oxygen (DO)^6^. Despite being tolerant to temperature deviation, any alterations outside their ideal rearing range (25–28 °c), could adversely weaken the fish’s antioxidant activity and inhibit the response of their immune system, either cell-mediated or humeral immunity^7,8^.

Owing to their impacts on the natural ecology, climate change, and global warming have been a significant issue for researchers^9^ and they will continue to be one of the greatest risks to aquatic animals ' vitality for quite a while^10–12^. This phenomenon has been associated with multiple adverse impacts including, altered fish availability and geographic distribution^13^, impaired metabolism^14,15^, enhanced spread and multiplication of pathogenic microorganisms. These impacts lead to a severe depletion in oxygen, and nutrition sources, rendering fish easy prey for stress and diseases^16–18^. Hence, all the aforementioned impacts combined end up with retarded growth and serious mortalities^19,20^. As a response to exposure to high thermal stress conditions, the activities of various fish proteins including, immunoglobulins, lysozyme, and complement are markedly inhibited^21,22^.

Recently, *Spirulina platensis* (SP) feed additives proved their promising impacts in the aquaculture field with potent antioxidant properties^23,24^. These properties could be imputed to its content of unique antioxidant components like phycocyanin, tocopherols, superoxide dismutase enzyme, and carotenoids, wholly effective in scavenging free radicals^25^. *S. platensis* also contains ideal amounts of fucoxanthin, chlorophyll, polysaccharides, vitamins E, and C, and polyphenols. These substances possess potent immunostimulant activities by enhancing the activities of the lysozyme complement system, phagocytic cells, and white blood cells (WBCs)^26,27^. The anti-inflammatory features, qualify *S. platensis* to exert resistance to infectious disease with extended protection to the liver and kidney^28^, thus, providing better mucosal immunity^27,29^. Along with its antiviral and anticancer properties, it also exhibits promising effects against anemia, heavy metal toxicity, obesity, diabetes, hyperlipidemia, and inadequate nutrition^30–32^. By absorbing nitrites and heavy metals, *S. platensis* also improves water quality and subsequently fish performance and survivability^33^. Dietary *Spirulina platensis* has been identified in multiple studies as a promising option to enhance gut microbiota, immunological and biochemical indices, and growth progress in various fish species^34,35^.

Dietary Coenzyme Q10 (Co-Q10) is noted as an effective scavenging agent for free radicals, naturally found in most living cells’ mitochondria^36^. Co-Q10 not only promotes vitamin regeneration, but also protects various cells (DNA, cell membranes, proteins, and lipids) from oxidative damage^37^. Moreover, it has a powerful action as an oxidative stress- alleviating agent and, hence, efficiently aids in mitigating oxidative stress in various species of fish; *Oreochromis niloticus*^38,39^, *Clarias gariepinus*^40^, *Oncorhynchus mykiss*^41^, *Liza ramada*^42^, *Dicentrachus labrax*^43^. Interestingly, Co-Q10 exerts an essential action in augmenting host durability against diseases and the general health of fish through modulating immunological responses^44^. At the tissue level, Co-Q10 can enhance metabolism and nutritional absorption^45,46^. As an issue, it is generated at an insufficient level in the body, so, this shortfall must be compensated by exogenous dietary supplements^47^.

The synergistic combination of *Spirulina platensis* and Coenzyme Q10 was strategically selected based on their complementary mechanisms of action in combating oxidative stress and enhancing immune function. While Spirulina provides a rich source of phycocyanin, β-carotene, and polysaccharides that directly scavenge free radicals and stimulate immune cell activity^23–27^, CoQ10 functions primarily as an electron carrier in the mitochondrial respiratory chain and a membrane-stabilizing antioxidant^36,37^. This combination addresses oxidative stress at multiple cellular levels: Spirulina neutralizes extracellular and cytoplasmic reactive oxygen species, while CoQ10 protects mitochondrial membranes and enhances cellular energy production^48,49^. Furthermore, the nanoemulsion delivery system was employed to overcome the limitations of both compounds—Spirulina’s relatively large particle size and CoQ10’s poor water solubility—thereby enhancing bioavailability, cellular uptake, and ultimately their therapeutic efficacy^50,51^. The nanoscale formulation facilitates penetration through the intestinal mucus layer and epithelial cells, maximizing absorption and distribution to target tissues^52,53^. This multi-level approach targeting both cytoplasmic and mitochondrial oxidative stress pathways is particularly relevant for heat stress conditions, where ROS generation is elevated across multiple cellular compartments^54,55^.

Numerous opportunities are presented by nanotechnology in the aquaculture and fisheries sectors. For instance, aquaculture species can benefit from improved fish feed made possible by nanotechnology. Multiple fields of aquaculture and fisheries can benefit from the novel materials created by nanoscience. For the different cells of the fish’s immune system, different nanoparticles are less damaging. As a unique and sensitive technique for identifying bacterial, fungal, and viral diseases in aquaculture, nanoparticles have attracted attention^56^. Likewise, aquaculture’s use of nanoparticles has demonstrated potential in enhancing drug delivery, illness diagnosis, aquatic animal nutrition, water quality, and management^57^.

Accordingly, this study aimed to evaluate spirulina-co-enzyme Q10 nanoemulsion (SCN) impacts as a novel, promising dietary inclusion on growth progress, immunity, stress-related profile, antioxidant indices, and tissue histoarchitecture of reared Nile tilapia in heat stress conditions.

## Materials and methods

### Synthesis and characterization of Spirulina-coenzyme Q10 nano emulsion (SCN)

Spirulina powder (*Spirulina platensis*) was produced from an alga unit in the National Research Center, Giza, Egypt. Coenzyme Q10 (Co-Q10) was purchased from Mepaco-Medifood Co. (Sharkia, Egypt). The Spirulina-coenzyme Q10 nanoemulsion was synthesized using a sonochemical method with a carefully optimized formulation ratio. The nano emulsion was prepared using olive oil as the oil phase, Tween 80 as the surfactant, and Spirulina-coenzyme Q10 as the active component in an aqueous medium. Before nanoemulsion synthesis, Spirulina powder (10 g) and Coenzyme Q10 (5 g) were pre-mixed and dispersed in 100 mL of distilled water under magnetic stirring at 500 rpm for 30 min at room temperature to ensure homogeneous distribution. This aqueous suspension of Spirulina-CoQ10 was then used as the active component phase during the nanoemulsion preparation. The pre-mixing step was essential to ensure the uniform distribution of both active ingredients throughout the final nanoemulsion matrix. The formulation ratio was maintained at 1:2:3 (olive oil: Tween 80: Spirulina-coenzyme Q10) to ensure optimal encapsulation efficiency and stability. The sonochemical synthesis was performed using a probe-type ultrasonicator (Model: Sonics Vibra-Cell VCX 750, Sonics & Materials Inc., USA) operating at a frequency of 20 kHz with a maximum power output of 750 W. The synthesis protocol involved: (1) pre-mixing olive oil and Tween 80 at room temperature (25 °C) for 10 min using magnetic stirring at 500 rpm, (2) adding the Spirulina-CoQ10 aqueous suspension dropwise while maintaining continuous stirring, (3) sonicating the mixture at 60% amplitude (approximately 450 W) for 15 min with pulse mode (5 s on, 2 s off) to prevent overheating, and (4) cooling the sample in an ice bath during sonication to maintain temperature below 30 °C. The total processing time was 15 min, resulting in a stable nanoemulsion with uniform particle size distribution. The sonochemical synthesis was performed using controlled parameters to achieve uniform particle size distribution and enhanced bioavailability of the active compounds.

#### Scanning electron microscopy (SEM) analysis

The morphological characterization and surface topology of the composite nanoparticles were investigated using scanning electron microscopy to visualize the nanoemulsion structure and particle distribution. SEM imaging was conducted using a Zeiss LEO Supra 55VP Field Emission SEM and SEM Zeiss 1530 SEM system. SEM imaging was performed at an accelerating voltage of 15 kV with magnification ranges of 50,000× to 120,000×, with the optimal visualization achieved at 120,000× magnification. The working distance was maintained at 8–10 mm to ensure optimal resolution and depth of field. Sample preparation involved diluting the composite nanoparticle suspensions 10-fold with their respective dispersion medium, followed by direct deposition of a droplet onto a polished aluminum sample holder. The samples were subsequently dried under vacuum conditions and coated with gold using an EMITECH K450X sputter coater to enhance conductivity and image quality.

#### X-ray diffraction (XRD) analysis

The crystalline structure and phase composition of the integrated nanoparticles were analyzed using X-ray diffractometry to determine the crystalline phase composition and degree of crystallinity of the nanoemulsion components. XRD analysis was performed using a D8-Find Bruker diffractometer equipped with CuKα radiation (λ = 1.5418 Å) operating at 40 mA current and 40 kV voltage with a step filter of 0.01°. Prior to measurement, dried samples were prepared using a planetary ball mill (LZQM0.4 L, Shicheng Desert Spring Mineral Gear Manufacturing Co., Ltd.), where stainless steel balls of 0.1 cm diameter were employed in the grinding process for 1 h at 1500 rpm to ensure homogeneous particle size distribution and improved crystalline structure analysis.

#### Dynamic light scattering (DLS) and zeta potential analysis

Dynamic light scattering analysis, also referred to as photon correlation spectroscopy (PCS) or quasi-elastic light scattering (QELS), was employed to determine the particle size distribution of the nanoemulsion in suspension. DLS measurements were performed using a Malvern Zetasizer Nano ZS instrument (Malvern Instruments Ltd., Worcestershire, UK) equipped with a 633 nm He-Ne laser at a fixed scattering angle of 173°. Before measurement, samples were diluted 100-fold with distilled water (dispersant medium) and equilibrated at 25 °C for 3 min. Three measurements were performed for each sample, with each measurement consisting of 12–15 sub-runs. The temperature was maintained at 25.0 ± 0.1 °C throughout the analysis using the instrument’s integrated Peltier temperature controller. The DLS technique utilizes the principle of Brownian motion, where smaller particles exhibit faster movement compared to larger particles in a liquid medium, allowing for accurate size distribution measurements. The scattered light intensity fluctuations resulting from random particle movement were analyzed using the Stokes-Einstein equation to determine particle size distribution.

#### Atomic force microscopy (AFM) analysis

Atomic force microscopy was utilized to obtain high-resolution topographic imaging and surface analysis of the nanoemulsion particles with demonstrated resolution on the order of fractions of a nanometer. AFM analysis provides three major capabilities: force measurement, topographic imaging, and manipulation, making it an essential tool for comprehensive nanoparticle characterization. AFM analysis was conducted using a commercial AFM system (NT-MDT NTEGRA, NT-MDT Spectrum Instruments, Russia) operating in semi-contact (tapping) mode to minimize sample damage while maintaining high resolution. Silicon cantilevers with a resonance frequency of 150 kHz and a spring constant of 5 N/m were employed. Multiple scan areas were examined, ranging from 0.50 × 0.50 μm to 5.0 × 5.0 μm, with the 0.50 × 0.50 μm area providing optimal visualization of individual nanoparticles. The scan rate was maintained at 0.5–1.0 Hz with 512 × 512-pixel resolution. The AFM system employs piezoelectric elements that facilitate precise and accurate movements on electronic command, enabling detailed scanning of the sample surface.

### Conditions for fish cultivation and the design of the experiment

A total of 225 mono-sex male Nile tilapia weighing 35.99 ± 0.59 g were acquired from Abbassa private fish farm at Abo-Hammad, Sharkia governorate, Egypt. Experimental trials were performed in the Department of Aquatic Animal Medicine’s wet lab, Faculty of Veterinary Medicine, Zagazig University, Egypt. Approximately two weeks ahead of the experiment’s commencement, fish became accustomed to laboratory settings in 100-liter glass tanks that were thermostatically managed and received dechlorinated tap water. The debris was siphoned once daily throughout the trial and acclimatization phases, and three weekly full water refills were conducted. Fish were subjected to a two week- acclimatization period, and the experimental design followed a completely randomized design (CRD) with five treatment groups, each containing three replicate tanks (*n* = 3), with 15 fish per tank (45 fish per treatment group), providing adequate statistical power for detecting treatment effects. The first group (C25) received baseline feed and represented the negative control. The second group (SCN0) served as positive control, it was raised at 32 °C and given a basal diet. The third to fifth groups were reared at 32 °C and were raised on varying concentrations of SCN: 10 (SCN10), 20 (SCN20), and 40 mg/kg diet (SCN40), respectively. The supplementation levels of 10, 20, and 40 mg/kg diet were selected based on previous literature demonstrating effective doses of *Spirulina platensis* (10–100 g/kg diet)^27,58,59^ and Coenzyme Q10 (20–80 mg/kg diet)^38,42,60^ in various fish species. The combined formulation was tested at lower concentrations (10–40 mg/kg) due to the enhanced bioavailability expected from the nanoemulsion delivery system. These doses were hypothesized to provide optimal antioxidant and immunostimulant effects while maintaining cost-effectiveness for practical aquaculture applications. The fish were fed 2 times daily (9:00 and 15:00) until they appeared satisfied. Heat stress conditions (32 ± 0.5 °C) were maintained constant throughout the 60-day experimental period using submersible thermostatically controlled aquarium heaters (300 W, Eheim Jager, Germany) installed in each experimental tank. Temperature was monitored continuously using digital thermometers (accuracy ± 0.1 °C) positioned at mid-water depth, with readings recorded three times daily (08:00, 14:00, and 20:00 h) to ensure temperature stability. The control group (C25) was maintained at 25 ± 0.5 °C using the same heating system with lower temperature settings. Water temperature in all tanks remained within ± 0.5 °C of target values throughout the experimental period, representing chronic constant heat stress conditions rather than cyclic thermal variation. With constant aeration, oxygen saturation was achieved. With permission number ZU-IACUC/2/F/240/2023, Zagazig University’s Animal Care and Research Use Authority has validated the methodology and design of the experiment.

### Diet preparation

Food preparation procedures were previously discussed by Ayyat et al.^61^. A 2 mm diameter pelleting machine was used to combine, homogenize, and pellet the components of the experimental diet. A homogeneous mist of spirulina-coenzyme nanoemulsion at varying concentrations was applied to the feed. To stop SCN degradation, the meals were dried at ambient temperature in a dark environment. The food contents and relative compositions employed throughout the experiment were carried out to satisfy Nile tilapia’s nutrient requirements^62^ (Table 1).

**Table 1:** Components and the experimental diets’ approximate content (g/kg).

Diet ingredients	Diets				
	C25	SCN0	SCN10	SCN20	SCN40
Fish meal (60% CP)	400	400	400	400	400
Soybean meal (44%)	200	200	200	200	200
Yellow corn	130	130	130	130	130
Wheat flour	150	150	150	150	150
Wheat Bran	20	20	20	20	20
Fish oil	70	70	70	70	70
Monocalcium phosphate	20	20	20	20	20
^(1)^ Vitamin mixture	4.5	4.5	4.5	4.5	4.5
^(2)^ Mineral mixture	5.5	5.5	5.5	5.5	5.5
spirulina co-enzyme nano-emulsion	0	0	0.01	0.02	0.04
Calculated composition (% DM)					
Crud Protein	38.90	38.90	38.90	38.90	38.90
Crude fat	10.50	10.50	10.50	10.50	10.50
Ash	5.84	5.84	5.84	5.84	5.84

* Control normal diet^138^.

^(1)^ Vitamin mix (IU or mg kg diet): vitamin D, 8000 IU; vitamin A, 16,000 IU; vitamin K, 14.72; vitamin B1, 17.8; vitamin B2, 48; vitamin B6, 29.52; cyanocobalamin, 0.24, tocopherols acetate, 160; vitamin C (35%), 800; niacinamide, 79.2; calcium-D- pantothenate,73.6; folic acid, 6.4; vitamin B7, 0.64 L-carnitine, 100.

^(2)^Mineral mix (mg kg diet): Cu (CuSO4), 2.0; Mn (MnSO4), 6.2; Zn (ZnSO4), 34.4; Fe (FeSO4), 21.1; I (Ca (IO3)2), 1.63; Co (CoCl2), 0.24; Se (Na2SeO3), 0.18; Mg (MgSO4.H2O), 52.7.

### Evaluation of growth performance parameters

The fish’s initial weights were recorded at the onset of the experiment. After 60 days, growth indexes, including weight gain (WG), condition factor (CF), feed conversion ratio (FCR), and specific growth rate (SGR), were assessed according to the following formula: BWG% = (FBW − IBW) ∕IBW**×** 100, SGR = 100 **×** (ln WT − lnWI)∕period∕day, and FCR = total feed intake (g)∕net gain (g). Condition factor (CF), as another health index, was also assessed: CF = W/L^3^** × **100. Survivability % was measured relying on El Basuini et al.^60^ study, using the following formula: Survival% = (count of survived fish at the end of the experiment period/ initial number of fish per group) **×** 100.

### Benefit–cost analysis

A number of economic metrics were computed to assess the experimental diets’ profitability. The mean total weight gain and the cost per kilogram of weight gain were multiplied to estimate the cost of manufacturing. The market price per kilogram of meat was multiplied by the average total weight gain to calculate the revenue. The difference between revenue and production costs was used to compute the gross margin. Lastly, the ratio of total returns to total expenses (BCR = total returns / total costs) was calculated to determine the benefit–cost ratio (BCR)^63^.

### Sampling of blood and tissue specimens

On the 60th day of the experiment, according to Neiffer and Stamper^64^, tranquilization of fish was done using a 100 mg/L benzocaine solution after fasting for 24 h. Using the caudal blood vessels technique, a pair of blood sets was extracted from each group of fish. To estimate the respiratory burst (NBT), lysozyme, and phagocytic activities in whole blood, the first set was collected via syringes containing heparin. The second set was aspirated in the absence of an anticoagulant for serum obtaining. Following that, samples for serum underwent a 15-minute centrifugation at 1500 rpm. To be used for immunity testing, the obtained serum samples were stored at -20 °C. Once that, the fish were euthanized by utilizing 400 mg L^− 1^ of benzocaine solution per the earlier protocol of Tran-Duy et al.^65^. Then, liver samples were aseptically excised and used for evaluation of antioxidant activity. For the histopathological procedures, specimens from the spleen, liver, and intestine were utilized.

### Hepatic oxidative stress biomarkers

Spectrophotometric methods were used to evaluate the hepatic oxidative stress biomarkers activity in the tissue homogenate. The procedure of homogenate preparation was previously documented by Rahman et al.^66^. The activity of the Glutathione-S-transferase (GST) enzyme was estimated using a substrate of 1-chloro-2-dinitrobenzene, following Habig et al.^67^. The superoxide dismutase (SOD) level was calculated using a colorimetric method by diagnostic kits of Catalog No.: SOD 25 21, Bio-diagnostics Co., Cairo, Egypt). Catalase (CAT) and malondialdehyde (MDA) activities were determined by using specific kits (Catalog No. MBS038818 and MBS007853, respectively) following the manufacturer’s recommendations (My BioSource, Inc., San Diego, CA 92195–3308, USA, respectively).

###  Immune indices assay

The activity of respiratory burst (RBA) was quantified in heparinized blood samples using the nitro-blue tetrazolium reduction (NBT; Sigma, USA) to formazan according to the assay mentioned by Anderson and Siwicki^68^ with some alterations in procedures as listed by Kumari and Sahoo^69^. The phagocytic activity (PA) was estimated following the techniques outlined by Kawahara et al.^70^. Following the addition of *Candida albicans* to the blood sample (A 50-mg per 1 ml of blood), a shaking step in a water bath at 25 °C for 5 h was performed. Giemsa stain was then applied to the blood smears. A randomly chosen 200 phagocytic cells engulfed a certain number of yeast cells and were utilized for phagocytic activity percentage estimation. The phagocytic percent (PA%) = No. Of macrophages with engulfed yeast/total No. Of macrophages × 100 Demers and Bayne^71^. The phagocytic index (PI) = the total number of engulfed yeast cells divided by the number of phagocytes with engulfed yeast^72^. Lysozyme action (LYZ) was measured utilizing a turbidimetric method relying on the lysis of *Micrococcus lysodeikticus* (Sigma-Aldrich, USA) freeze-dried particles^73^. Nitric oxide (NO) level in serum was estimated using the colorimetric assay outlined by Montgomery and Dymock^74^. Commercial kits (Catalog No. MBS281020) obtained from My BioSource Co. (California, USA), were employed to measure complement 3 (C3) in serum samples following the manufacturer’s protocol. IgM values were estimated using diagnostic kits of Catalog No. CSB-E1205Fh (Cusabio Co, Houston, TX, USA) following the recommendations of the manufacturer.

### Histopathological examination

The histopathological inquiries involved obtaining nine samples from each of the spleen, mid-portion of the gut, and liver, fixing them in 10% neutral buffered formalin, dehydrating them in increasing quantities of ethanol, cleaning them in xylene, and embedding them in paraffin. The sections were then stained with hematoxylin and eosin (H&E) using a microtome (Leica^®^, Wetzlar, Germany), and morphometric measurements and slide microscopic analysis were performed using a Ceti England microscope equipped with a digital camera (AmScope)^75^. All procedures were processed by employing the AmScopeToupView software version 3.7 (AmScope, United States), which included villus height (measured from tip to base), villus diameter (measured from medial area, side to side), goblet cell number, and submucosal layer thicknesses (per region at high magnification, i.e., 400x).

### Challenge study

After a 60-day experimental trial, 5 fish per replicate (15/group) were challenged by a virulent isolate of *S. agalactiae.* The utilized isolate was earlier isolated from diseased Nile tilapia and identified using phenotypic and molecular identification, then it was kept in BHI broth (brain heart infusion + 20% glycerol) and preserved at − 20 °C till use, Abdel Rahman et al.^76^. The determined LD_50_ of *S. agalactiae* in Nile tilapia was noted by a dose equal to 1 × 10^7^ CFU/mL. Via the intraperitoneal route of injection, fish received a sub-lethal dose of a 24-hour-old broth culture of *S. agalactiae* (0.2 ml of 0.5 × 10^6^ CFU/mL). A standard MacFarland tube was used for thorough adjustment of the bacterial inoculum concentration.

### Statistical assay

The values were examined for normal distribution using the Shapiro-Wilk test. Levene’s test was applied to check the homogeneity of variance before moving on to statistical evaluation. One-way analysis of variance, or ANOVA, was employed to statistically analyze the research data (SPSS Inc., Chicago, IL, USA). Duncan’s post hoc test, which was set at p˂0.05, was used to recognize significant changes between means. The findings were highlighted as means ± standard error (SE).

## Results

### SCN characterization results

**Fig. 1:**
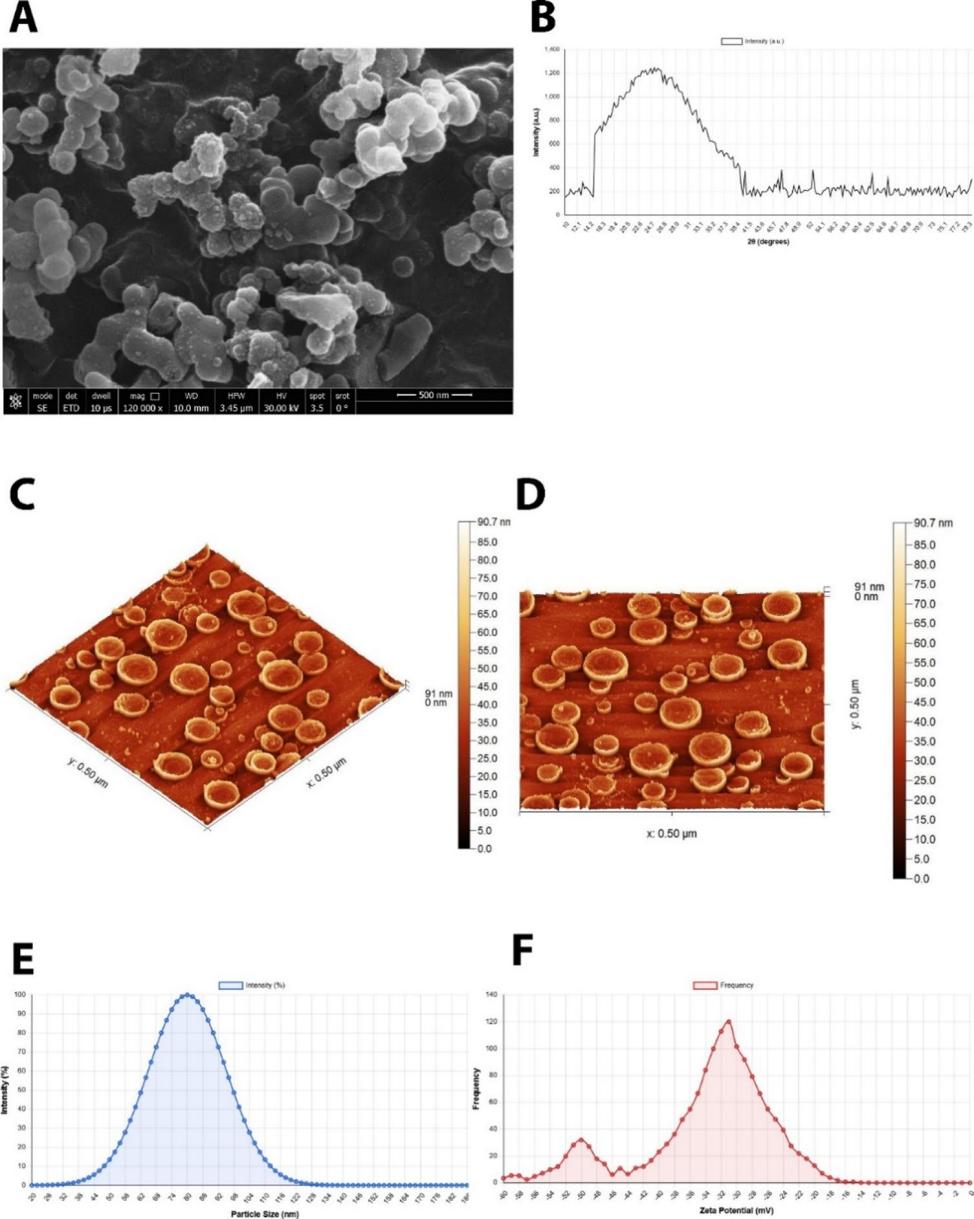
Comprehensive physicochemical characterization of Spirulina-coenzyme Q10 nanoemulsion (SCN) synthesized using sonochemical method with 1:2:3 ratio formulation. (**A**) Scanning electron microscopy (SEM) image displaying well-dispersed spherical nanoparticles with smooth surface morphology and uniform size distribution without aggregation (scale bar = 500 nm, magnification: 120,000×). (**B**) X-ray diffraction (XRD) pattern showing characteristic broad peak around 20–25° 2θ with maximum intensity of approximately 1,200 a.u., indicating amorphous/semi-crystalline structure typical of nanoemulsion systems. (**C**) Three-dimensional atomic force microscopy (AFM) topographic image revealing spherical nanoparticles with height range of 0.0–90.7 nm distributed across 0.50 × 0.50 μm scan area, demonstrating nanoscale dimensions and uniform particle morphology. (**D**) Two-dimensional AFM height image providing detailed visualization of individual spherical particles with consistent size and well-separated distribution, confirming successful nanoparticle formation. (**E**) Dynamic light scattering (DLS) intensity distribution curve exhibiting unimodal bell-shaped profile with approximately 100% intensity at peak, indicating narrow particle size distribution and high uniformity. (**F**) Particle size frequency distribution obtained from DLS analysis demonstrating narrow size distribution with highest frequency in the nanometer range, confirming excellent size uniformity and low polydispersity of the nanoemulsion system.

The comprehensive physicochemical characterization revealed the following quantitative parameters: Dynamic light scattering analysis (Malvern Zetasizer Nano ZS, Malvern Instruments Ltd., UK) showed an average hydrodynamic diameter of 187.3 ± 8.5 nm with a narrow size distribution and polydispersity index (PDI) of 0.21 ± 0.02, indicating excellent size uniformity. The zeta potential measurement yielded a value of -28.4 ± 1.8 mV, confirming adequate surface charge for long-term colloidal stability through electrostatic repulsion. The negative zeta potential is attributed to the presence of carboxyl and phosphate groups from Spirulina components. Encapsulation efficiency, determined by centrifugation and spectrophotometric analysis, was 89.3 ± 2.1% for Spirulina (measured at 620 nm based on phycocyanin content) and 91.7 ± 1.9% for CoQ10 (measured at 275 nm), demonstrating high retention of both active compounds within the nanoemulsion matrix. The scanning electron microscopy analysis presented in Fig. 1A revealed the successful formation of spherical nanoparticles with relatively uniform size distribution throughout the nanoemulsion matrix. The X-ray diffraction analysis presented in Fig. 1B displays a characteristic broad peak with maximum intensity around 20–25°, indicating the presence of an amorphous or semi-crystalline state within the lipid matrix. The atomic force microscopy analysis provided detailed three-dimensional topographic information about the nanoemulsion particles at the nanoscale level. Figure 1C presents a three-dimensional AFM topographic image revealing spherical particles with uniform height distribution across a 0.50 × 0.50 μm scan area. The height scale ranging from 0.0 to 90.7 nm demonstrates the nanoscale dimensions of the particles, with individual particles clearly visible as elevated circular structures on the substrate surface. The AFM data confirms the morphological observations from SEM analysis, showing smooth spherical particles without surface irregularities or aggregation. Figure 1D displays the corresponding two-dimensional AFM height image, providing a detailed visualization of individual particle morphology and distribution. The image clearly shows well-separated spherical nanoparticles with consistent size and shape, indicating successful formation of individual nanoparticles with controlled dimensions. The dynamic light scattering analysis (DLS) results presented in Fig. 1E revealed a narrow particle size distribution with optimal dimensions for oral bioavailability and cellular uptake. The intensity distribution curve shows a unimodal distribution with a characteristic bell-shaped profile, demonstrating the predominance of particles within the nanoscale range and confirming successful nanoemulsion formation. Figure 1F presents the frequency distribution obtained from DLS analysis, showing a narrow particle size distribution with the highest frequency occurring in the nanometer range. The frequency distribution demonstrates excellent size uniformity with minimal polydispersity, which is crucial for consistent therapeutic performance. The measured zeta potential values indicated sufficient surface charge to maintain electrostatic repulsion between particles, preventing aggregation and ensuring long-term stability of the formulation. The negative surface charge observed is consistent with the presence of Spirulina extract components and contributes to the overall stability of nanoemulsion through electrostatic stabilization mechanisms.

### Survivability and growth performance evaluation

Growth indices were illustrated in Table 2. Overall, SCN supplementation enhanced the different growth indicators in heat-stressed Nile tilapia. Fish-fed SCN20 and SCN40-enriched diets displayed significant improvement (*p* < 0.05) in WG and SGR compared to the SCN0 and showed an improvement approaching the C25 groups. However, FCR showed a notable reduction in SCN20 and SCN40 groups, while SCN0 expressed the highest value. The SC40 group exhibited a higher survival rate (93.33%), followed by the SCN20 group (91.1%), whereas the lowest records were found in the SCN0 group (66.66%).

**Table 2:** The effect of spirulina-Co-enzyme Q10 nanoemulsion-fortified diet on the growth progress in heat-stressed Nile tilapia for 60 days.

Parameters	Experimental groups						*P*-value
	C25		SCN0	SCN10	SCN20	SCN40	
Initial body weight (g)	36.16 ± 0.72		36.26 ± 0.37	36.23 ± 0.39	36.56 ± 0.43	36.04 ± 0.29	0.978
Final body weight (g)	78.66 ± 0.88^a^		51.66 ± 1.6^d^	64.00 ± 2^c^	70.00 ± 0.00^b^	73.00 ± 1^b^	0.000
Total weight gain	42.5 ± 0.28^a^		16.66 ± 0.88^d^	27.76 ± 1.92^c^	33.76 ± 0.39^b^	36.43 ± 0.86^b^	0.000
Total feed intake	86.8 ± 1.74		84.00 ± 2.77	86.96 ± 0.94	86.96 ± 0.94	87.76 ± 1.04	0.569
SGR (%)	1.29 ± 0.01^a^		0.58 ± 0.03^d^	0.94 ± 0.05^c^	1.09 ± 0.01^b^	1.15 ± 0.02^b^	0.000
FCR	2.04 ± 0.03^c^		5.06 ± 0.39^a^	3.16 ± 0.05^b^	2.57 ± 0.05^bc^	2.41 ± 0.06^c^	0.000
CF	1.73 ± 0.05^a^		1.48 ± 0.001^b^	1.61 ± 0.08^ab^	1.65 ± 0.05^ab^	1.67 ± 0.04^ab^	0.098
SR %	97.77 ± 2.22^a^		66.66 ± 3.84^c^	77.77 ± 2.22^b^	91.10 ± 2.22^a^	93.33 ± 0.00^a^	0.000

SGR%; specific growth rate%, FCR, feed conversion ratio, CF; condition factor, SR; survival rate. The mean ± SE is used to express values. At *P* < 0.05 (one-way ANOVA; Duncan’s post hoc test), the means in the same row with distinct superscripts are significant (*n* = 9/group). C25 was raised at 25 ◦C, while SCN0, SCN10, SCN20, and SCN40 groups were raised at 32 ◦C.

### Results of the benefit–cost ratio (BCR)

As shown in Table 3, both SCN20 and SCN40 exhibited higher feed and total production costs than the negative control group (C25). Feed costs increased from 0.129 USD/kg gain in the C25 group to 0.137 and 0.145 USD/kg gain in SCN20 and SCN40, respectively. Similarly, total production costs rose from 0.172 USD/kg gain in the C25 group to 0.179 and 0.188 USD/kg gain in SCN20 and SCN40. Despite these higher costs, total returns in SCN20 (0.194 USD/fish) and SCN40 (0.202 USD/fish) remained relatively close to that of C25 (0.218 USD/fish). The gross margin decreased from 0.046 USD/fish in the C25 group to 0.014 USD/fish in both SCN20 and SCN40, indicating a moderate reduction in profitability. Correspondingly, the benefit–cost ratio (BCR) declined from 126.65 in the C25 group to 107.90 and 107.29 in SCN20 and SCN40, respectively.

**Table 3:** The benefit–cost analysis of the different dietary formulations.

Parameter	C25	SCN0	SCN10	SCN20	SCN40	SEM
Feed cost (USD/kg gain)	0.129^3bc^	0.125^a^	0.133^bc^	0.137^b^	0.145^a^	0.002
Total cost (USD/kg gain)	0.172^bc^	0.168^a^	0.176^bc^	0.179^b^	0.188^a^	0.002
Total return (USD/fish)	0.218^a^	0.143^d^	0.177^c^	0.194^b^	0.202^b^	0.006
Gross margin (USD/fish)	0.046^a^	–0.025^d^	0.0013^c^	0.014^b^	0.014^b^	0.005
Benefit–cost ratio (BCR)	126.65^a^	85.22^d^	100.71^c^	107.90^b^	107.29v	3.62

The costs of the formulated diets; basal diet: 1.48 USD/kg, SCN10: 153 USD/kg, SCN20:1.57 USD/kg, SCN40:1.65 USD/kg. The cost to the customer per kilogram of Nile tilapia: 2.74 USD. Total fixed cost 0.042 USD.

### Oxidative stress and lipid peroxidation biomarkers

The hepatic antioxidant indicators, including SOD, GST, and CAT, showed a significant reduction in SCN0 compared to the C25 group. SCN40 showed a non-significant difference in the activity of SOD and GST with the C25 group. The dietary supplementation of SCN to Nile tilapia has significantly raised (*p* < 0.05) the level of antioxidant indicators in a concentration-related pattern, compared to the heat-stressed, non-fortified group (SCN0). Nonetheless, the lipid peroxidation marker (MDA) displayed a marked decline in C25, SCN20, and SCN40 groups, relative to its higher level in SCN0 groups (Table 4).

**Table 4:** The effect of spirulina-Co-enzyme Q10 nanoemulsion-fortified diet on antioxidant indices, and lipid peroxidation marker in Nile tilapia reared under heat stress conditions for 60 days.

Parameters	Experimental groups					*P*-value
	C25	SCN0	SCN10	SCN20	SCN40	
CAT (u/g tissue)	9.87 ± .36^a^	4.4 ± 0.25^e^	5.91 ± 0.11^d^	6.94 ± 0.17^c^	8.79 ± 0.12^b^	0.000
SOD (u/g tissue)	208.63 ± 7.2^a^	96.02 ± 3.5^d^	139.94 ± 4.9^c^	160.5 ± 0.34^b^	197.7 ± 3.8^a^	0.000
GST (u/g tissue)	5.81 ± 0.3^a^	2.38 ± 0.07^d^	2.99 ± 0.06^c^	4.52 ± 0.18^b^	5.80 ± 0.17^a^	0.000
MDA (nmol/g tissue)	0.65 ± 0.06^d^	3.6 ± 0.06^a^	2.6 ± 0.07^b^	0.78 ± 0.06^c^	0.59 ± 0.14^d^	0.000

SOD: superoxide dismutase; CAT: catalase; GST: glutathione s transferase; MDA: malondialdehyde. The average values of three aquariums per treatment are represented by the treatment means. For every parameter, the means are displayed. Significant differences (P ˂ 0.05) exist between means in the same column with different superscripts. values are represented as the mean ± SE. The means within the same row carrying different superscripts are significant at *P <* 0.05 (One-way ANOVA; Duncan’s post hoc test) (*n* = 9/group).

### Immune biomarkers

The RBA was markedly boosted (*p* < 0.05) in C25 and all groups that received SCN, an incorporated diet, relative to the SCN0 group, which revealed the lowest RBA activity. The highest records of PA% and PI were evident in the C25 group, which was reared under a normal temperature range. A level-dependent elevation in both PA and PI was noted in SCN-fortified groups without a significant variance between SCN20 and SCN40 groups. Similarly, the activity of lysozyme, complement 3, NO, and IgM showed an ascending rise, which was associated with increasing the SCN supplementation level. Interestingly, a non-significant difference was recorded in both lysozyme and C3 levels between the C25 and SCN40 groups (Table 5).

**Table 5:** The effect of spirulina-Co-enzyme Q10 nano emulsion-fortified diet on immunological indicators of Nile tilapia reared under heat stress conditions for 60 days.

Parameters	Experimental groups					*P*-value
	C25	SCN0	SCN10	SCN20	SCN40	
NBT (mg/ml)	0.37 ± 0.00^a^	0.23 ± 0.00^c^	0.25 ± 0.00^b^	0.35 ± 0.00^ab^	0.35 ± 0.01^ab^	0.000
PA %	20.5 ± 0.86^a^	10.5 ± 0.28^d^	12.5 ± 0.28^c^	18.5 ± 0.57^b^	18.5 ± 0.00^b^	0.000
PI	1.14 ± 0.00^a^	1.03 ± 0.00^d^	1.08 ± 0.00^c^	1.11 ± 0.00^b^	1.13 ± 0.00^b^	0.000
Lysozyme	4.1 ± 0.08^a^	2.6 ± 0.15^d^	3.02 ± 0.08^c^	3.46 ± 0.05^b^	3.98 ± 0.13^a^	0.000
C3	28.31 ± 0.73^a^	15.25 ± 0.64^d^	20.24 ± 0.17^c^	24.45 ± 0.64^b^	29.57 ± 0.57^a^	0.000
NO	32.78 ± 0.89a	19.66 ± 0.32d	26.21 ± 0.38c	27.23 ± 0.31c	29.05 ± 0.11b	0.000
IgM	300.5 ± 0.28a	117.47 ± 6.4d	164.46 ± 4.5c	245.39 ± 6.3b	297.62 ± 1.37a	0.000

phagocytic activity% (PA%), phagocytic index (PI), lysozyme, complement 3 (C3), nitric oxide (NO), and IgM. For values, the mean ± SE is used. Duncan’s post hoc test (*n* = 9/group) indicates that means with distinct superscripts within the same row are significant at *P* < 0.05 (one-way ANOVA).

### Histopathological findings

#### Spleen

**Fig. 2:**
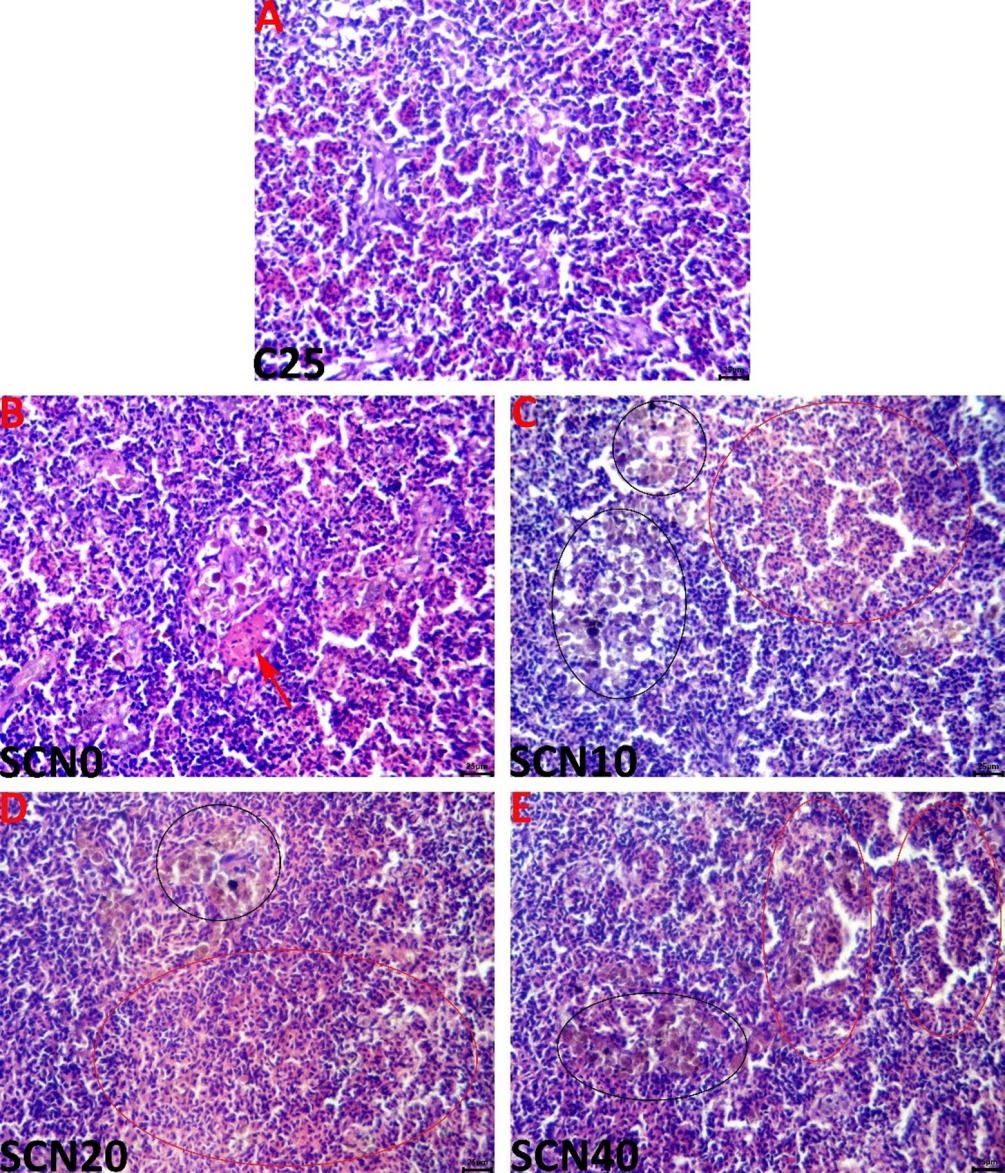
The C25 group exhibits normal histological images in representative light micrographs of the H&E-stained splenic tissue slices (**A**).and varying degrees of hypercellularity particularly of the erythroid elements (red ellipses), vascular congestions (red arrows), and MMCs hyperplasia (black ellipses) in the SCN0 (**B**), SCN10 (**C**), SCN20 (**D**), and SCN40 (**E**) groups. There is a 25 μm scale bar.

The spleens of the C25 group showed normal histological pictures (Fig. 2A). The effect of high-water temperature on the histology of tilapias was noticeable as the spleens of the SCN0 group showed hypercellularity, particularly in the erythroid elements, vascular congestion, and a considerable increase in the sizes of the MMCs (Fig. 2B). Similar but pronounced reactions were seen in the spleens of the tilapias fed on diets fortified by spirulina Co-enzyme Q10 nano-emulsions with almost no critical differences between the SCN10 group (Fig. 2C), the SCN20 group (Fig. 2D), and the SCN40 group (Fig. 2E). The assessed histopathological criteria in the splenic tissues of all groups are represented in Table 6.

**Table 6:** The effect of spirulina-Co-enzyme Q10 nanoemulsion-fortified diet on the histoarchitecture of the splenic, hepatic, and intestinal tissues in heat-stressed Nile tilapia for 60 days.

Organ	Histopathological criteria	C25	SCN0	SCN10	SCN20	SCN40	*P*-value
Liver	Vascular congestion	0.00 ± 0.00^b^	9.00 ± 2.33^a^	4.00 ± 1.63^b^	2.00 ± 1.33^b^	2.00 ± 1.33^b^	0.002
	Percentages of the area fraction occupied by hepatocytes’ cytoplasmic vacuolation um^2^	65.56 ± 1.4^a^	9.63 ± 1.45^d^	18.06 ± 2.07^c^	48.99 ± 0.80^b^	49.15 ± 1.25^b^	0.000
	Percentages of the area fraction occupied by MMCs um^2^	2.14 ± 0.24^c^	8.77 ± 0.40^a^	7.44 ± 0.23^b^	6.57 ± 0.29^b^	6.7 ± 0.416^b^	0.000
Spleen	Vascular congestion	0.00 ± 0.00^c^	15.00 ± 1.66^a^	6.00 ± 1.63^b^	4.00 ± 1.63^bc^	4.00 ± 1.63^bc^	0.000
	Erythroid hyperplasia	0.00 ± 0.00^b^	63.00 ± 4.48^a^	56.00 ± 2.66^a^	54.00 ± 3.05^a^	45.00 ± 3.05^a^	.000
	Lymphoid hyperplasia	0.00 ± 0.00^b^	13.00 ± 1.52^a^	12.00 ± 1.33^a^	12.00 ± 1.33^a^	14.00 ± 1.63^a^	0.000
	Percentages of the area fraction occupied by MMCs um^2^	4.56 ± 0.37^c^	8.8 ± 0.16^b^	9.7 ± 0.58^ab^	10.58 ± 0.87^a^	10.95 ± 0.85^a^	0.000
Intestine	Villus width um	115.8 ± 4.62^a^	90.5 ± 6.11^b^	100.6 ± 4.48^ab^	109 ± 3.81^a^	106.6 ± 6.23^a^	0.000
	Villus height um	705.3 ± 35.19^a^	479.8 ± 41.35^c^	541 ± 4992^bc^	649.4 ± 22.61^ab^	642.6 ± 24.71^ab^	0.016
	Villus surface areas um^2^	82099.7± 5887.05^a^	44830.2± 6983.01^c^	56258.9± 8804.06^bc^	70,695± 3276.65^ab^	68293.8± 4561.15^ab^	0.001
	Thickness of Lamina propria um	53.2 ± 4.46	55.1 ± 3.91	55.6 ± 3.96	55.00 ± 4.16	54.8 ± 4.26	0.996
	Thickness of tunica muscularis um	70.9 ± 7.44a	48.3 ± 5.61b	63.00 ± 4.77^ab^	69.9 ± 5.73^a^	70.8 ± 5.33^a^	0.040
	Villus bending	0.00 ± 0.00b	6.00 ± 1.63a	3.00 ± 1.52^ab^	2.00 ± 1.33^ab^	2.00 ± 1.33^ab^	0.037
	Epithelial desquamation	1.00 ± 1.00b	6.00 ± 1.00a	3.00 ± 1.63^ab^	2.00 ± 1.52^ab^	2.00 ± 1.33^ab^	0.121
	Inflammatory cell infiltrate	0.00 ± 0.00b	6.00 ± 1.63a	4.00 ± 1.63^ab^	2.00 ± 1.33^ab^	3.00 ± 1.52^ab^	0.046

The mean ± SE is used to express values. At *P* < 0.05 (one-way ANOVA; Duncan’s post hoc test), the means in the same row with distinct superscripts are significant (*n* = 9/group).

#### Liver

**Fig. 3:**
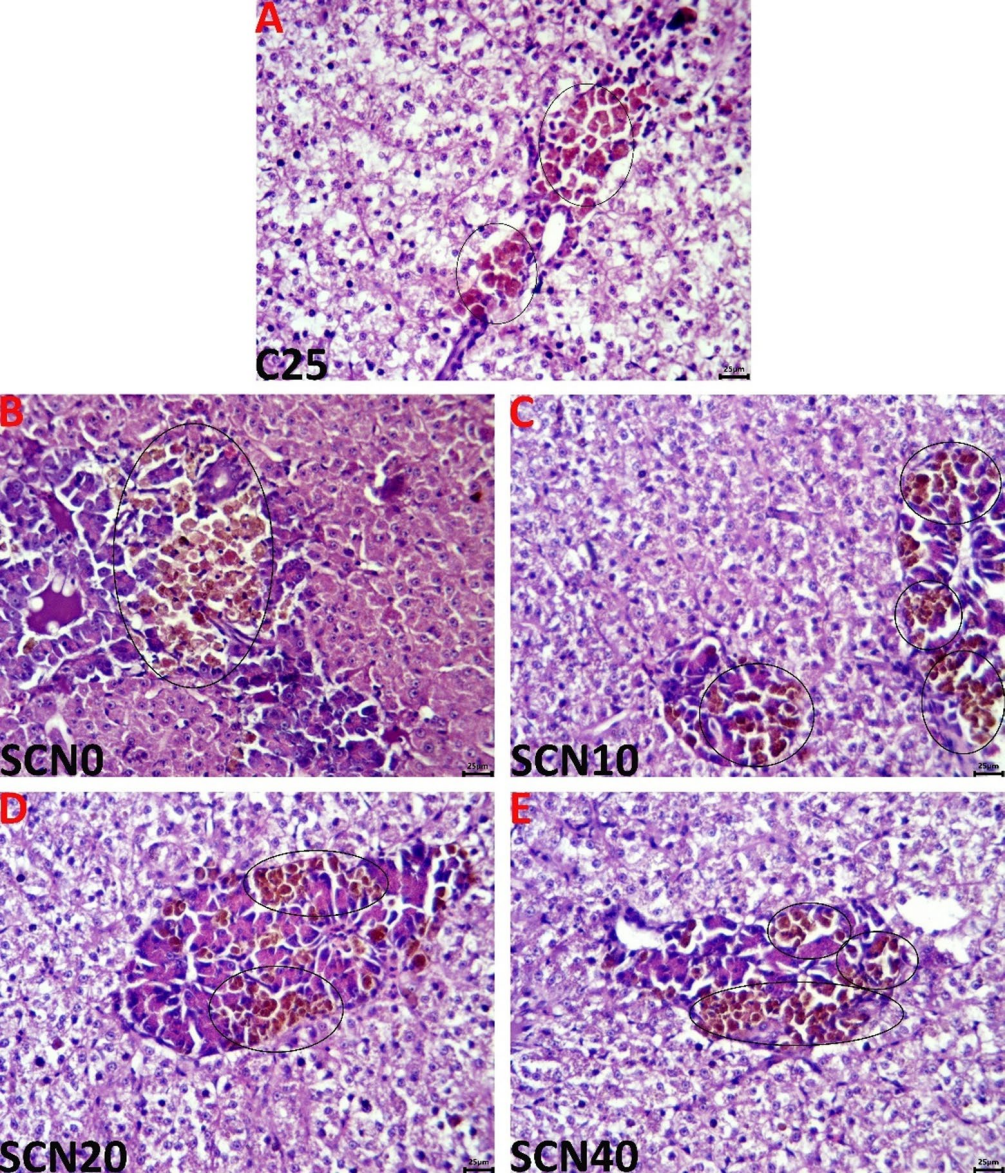
In the C25 group (**A**), representative light micrographs of the H&E-stained hepatic tissue sections display typical histological images; in the SCN0 group (**B**), there is a noticeable decrease in the cytoplasmic vacuolations of the hepatocytes together with a considerable increase in the diameters of the MMCs (black ellipses), and a dimension of these alterations at varying degrees in the SCN10 (**C**), SCN20 (**D**), and SCN40 (**E**) groups. The scale bar is 25 μm.

The C25 group liver samples revealed normal histology (Fig. 3A). High water temperature incited two main responses in the SCN0 group hepatic tissue, represented by a severe decrease in the hepatocytes’ cytoplasmic vacuolations associated with a vital increase in the sizes of the MMCs (Fig. 3B). Adding spirulina Co-enzyme Q10 nano-emulsions to the heat-stressed tilapia diet diminished the heat-stress-induced histopathological responses moderately in the SCN10 group (Fig. 3C), and significantly in the SCN20 (Fig. 3D), and the SCN40 group (Fig. 3E). The assessed histopathological criteria in the hepatic tissues of all groups are represented in Table 6.

#### Intestine

**Fig. 4:**
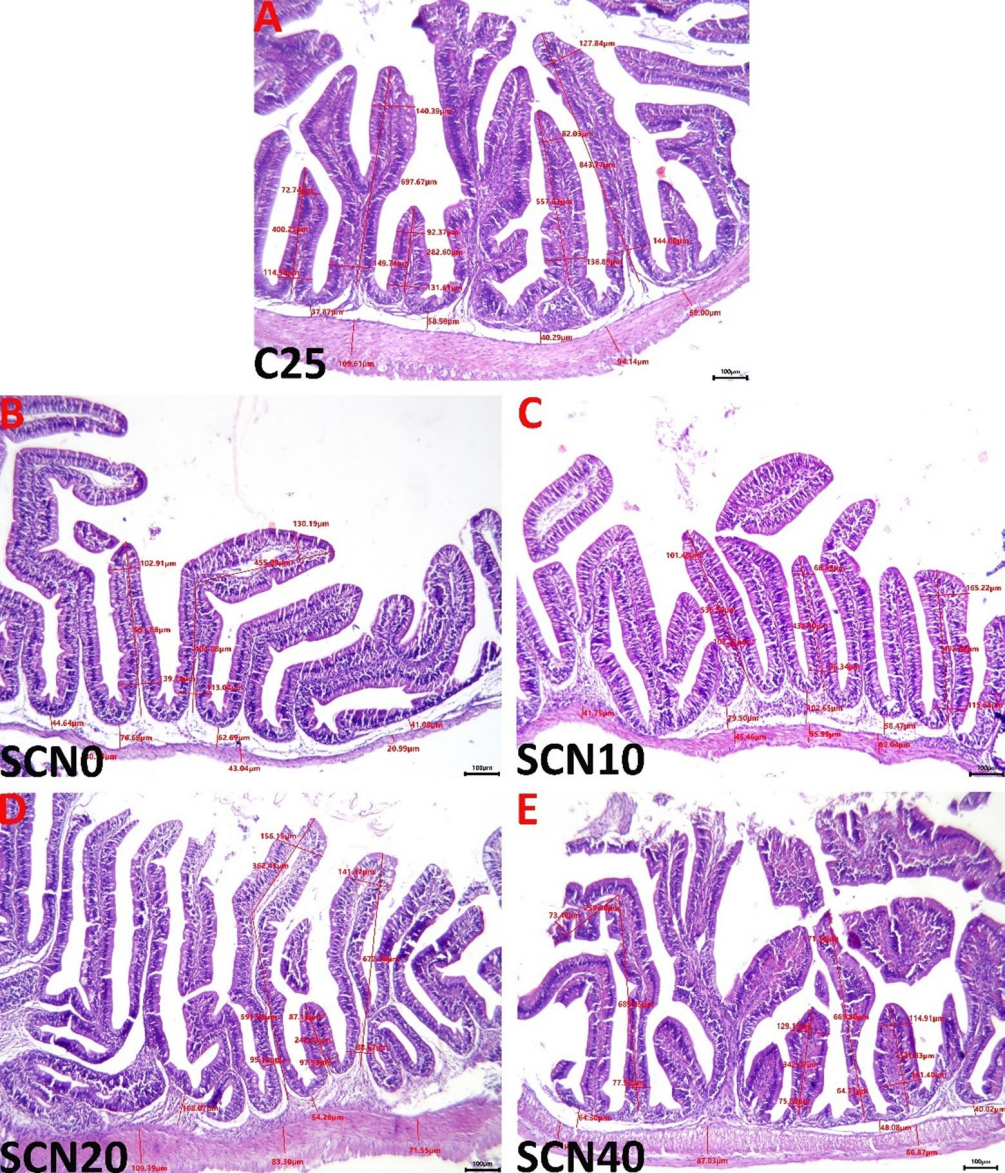
Representative light micrographs of the H&E-stained intestinal tissue sections show normal histological pictures in the C25 group (**A**), and villus bending with a reduction in the villus heights, widths, and thicknesses of the tunica muscularis, increased inflammatory cell infiltrates, and villus distortion in the CSN0 group (**B**). Moderate ameliorations of the heat water stress-induced alterations are seen in the SCN10 group (**C**), while significant ameliorations are observable in both the SCN20 (**D**) and SCN40 (**E**) groups. The scale bar equals 100 μm.

The C25 group intestinal specimen showed normal histological pictures (Fig. 4A). Rearing tilapia at 32 °C for 60 days adversely affected the intestinal histology in the SCN0 group by (1) reducing the villus widths, heights, surface areas, and thicknesses of the tunica muscularis, and (2) inciting degenerative and inflammatory responses, including increased inflammatory cell infiltrates, villus distortion, and epithelial desquamation (Fig. 4B). Adding spirulina Co-enzyme Q10 nano-emulsions to the diet of heat-stressed tilapia demonstrated notable entero-protective effects, especially at the 20 and 40 mg doses, and to a lesser extent at the 10 mg dose. The images representing the SCN10, SCN20, and SCN40 groups are shown in (Figs. 4C, D, and E), and all groups’ statistical data of the histopathological parameters of the intestinal tissues are listed in Table 6.

### Ethological, clinical, post-mortem disorders following *S. agalactiae* challenge

**Fig. 5:**
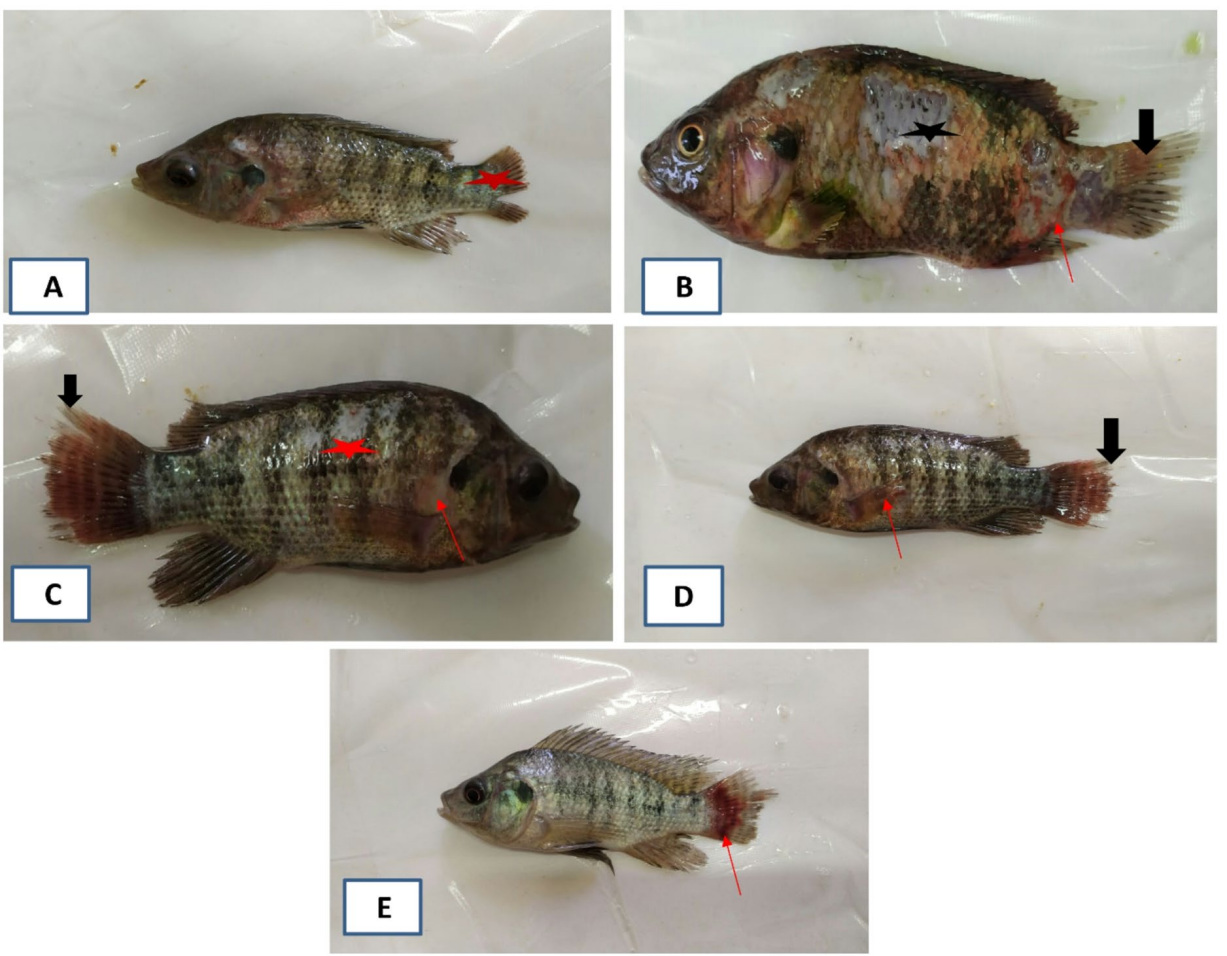
Clinical signs of *S. agalactiae* challenged- Nile tilapia. (**A**) Fish in the C25 group showing severe rot of caudal find (star). (**B**) Fish in the SCN0 group showing severe rot of dorsal and caudal fins (black arrow), hemorrhage, and deep ulcer at the peduncle area (red arrow) and superficial ulcer on the trunk region (star). (**C**) Fish in the SCN10 group show moderate fin rot, especially the caudal and dorsal ones (black arrow), localized detachment of scales (star), and erythema at the opercular margin (red arrow). (**D**) SCN20 group showing and moderate degree of fin rot (black arrow) and hemorrhagic pectoral fin (red arrow). (**E**) The SCN40 group expressed better health conditions compared to all stressed groups and exhibited caudal fin hemorrhage and rot (red arrow).

**Fig. 6:**
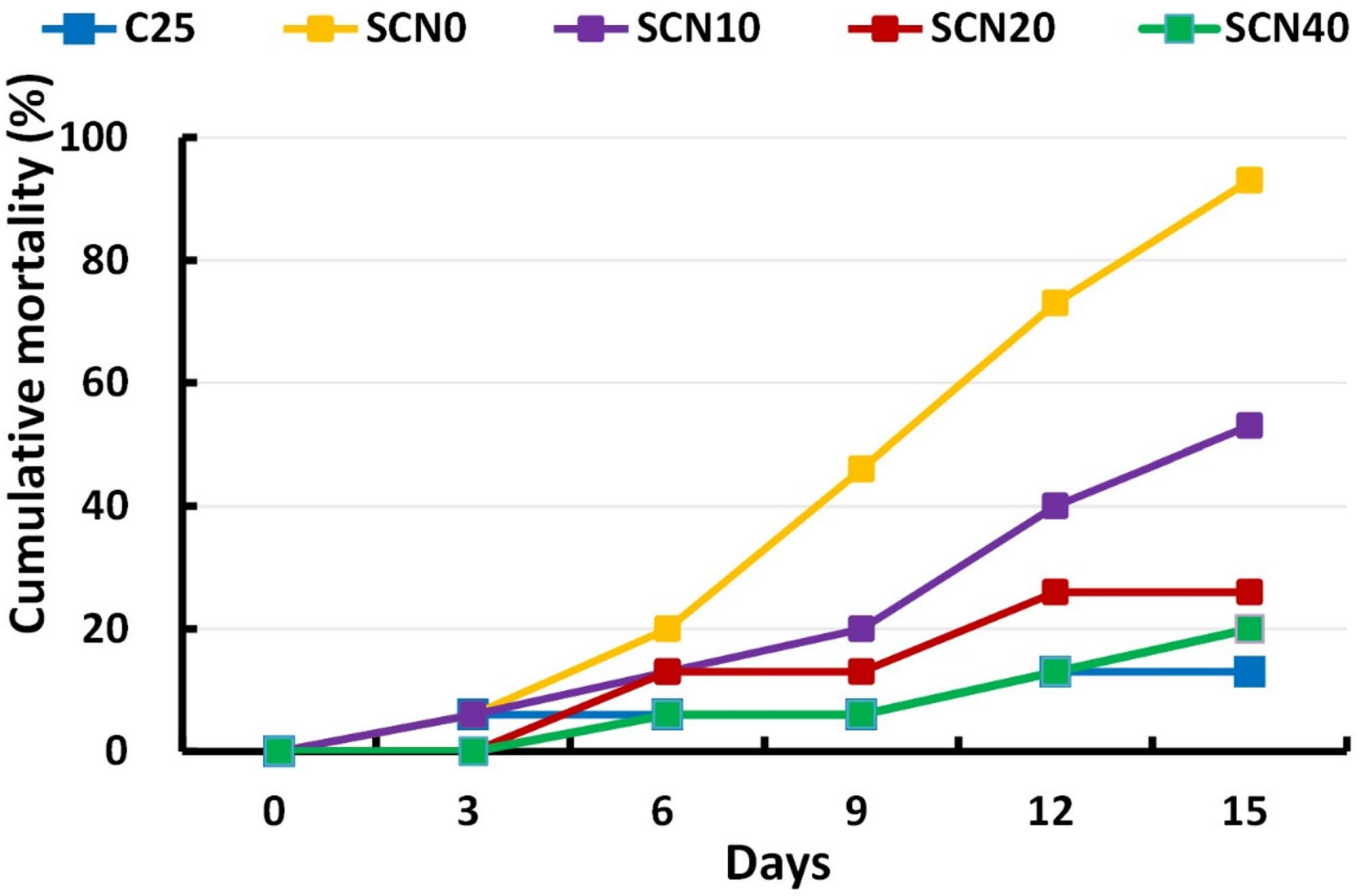
Cumulative mortality of *S. agalactiae-*challenged- fish after 14 days. SCN0, SCN10, SCN20, and SCN40 groups were reared at 32 °C, and C25 was reared at 25 °C.

As the experimental challenge progressed, the fish in the C25 group displayed insignificant behavior alterations with moderate clinical indications such as fin rot, hyperemia and inflammation at the site of bacterial inoculation (Fig. 5A). They also had the lowest cumulative mortality rate (13%) (Fig. 6). After the second day of infection, SCN0 exhibited the most severe clinical symptoms, which included severe fin rot, hemorrhages throughout the fish body, a slight protrusion of the eye with turbidity and hemorrhage in some cases. Other signs were also expressed as loss of scales, deep skin ulceration, and hemorrhagic anal opening (Fig. 5B). The ethological disorders displayed abnormal swimming behavior, either sluggishness, hyperactivity, or spiral movement. This group also possessed the highest mortality rate (93%). It is worth noting that numerous fish died suddenly without any discernible medical symptoms. Via the necropsy procedures, several organs revealed abnormal conditions due to internal congestion, where the spleen was enlarged, also the liver and gall bladder were affected. Nevertheless, compared to SCN0, SCN10 displayed less severe symptoms and a lowered cumulative death rate (53%) (Fig. 5C). Fin rot was one of the few or nonexistent symptoms displayed by SCN20 (Fig. 5D), and SCN40 (Fig. 5E), which had cumulative death rates of 26% and 20%, respectively.

## Discussion

Ecological elements, particularly temperature, are regarded as critical issues in the aquaculture industry. Extreme heat stress negatively impacts feed intake, growth development, oxidative status, and physiological activity, moreover, thefish’s immune systems are less equipped to fend against infections^77,78^. Commercial aquaculture species’ growth and survival are greatly influenced by temperature, which also has a direct effect on their immunological response, metabolic activity, and oxygen consumption. Higher temperatures within tolerance limits accelerate growth by increasing the metabolic rate, as demonstrated by the thermal growth index, which shows the relationship between temperature and growth development^79^. Fish that are subjected to temperature fluctuations beyond their thermal tolerance will stop feeding and show signs of diminished resistance^80^. Nowadays, the use of nanoemulsions in aquafeeds has attracted a lot of attention worldwide due to their positive effects on stress reduction, increased resistance to disease, and general health enhancement^50^. For the first time, our study proves that adding SCN to the Nile tilapia diet ameliorates the negative consequences of heat stress.

The particle size observed in the SEM image appears to be in the nanometer range, which is consistent with the nanoemulsion classification and optimal for pharmaceutical applications^81,82^. The broadening of the diffraction peak observed is characteristic of nanoscale materials and confirms the reduction in particle size achieved through the sonochemical synthesis method. This peak broadening, known as the Scherrer effect, indicates successful nanoparticle formation and correlates with the particle size data obtained from other characterization techniques. The intensity profile shows a gradual decrease from the maximum peak, suggesting a well-formed nanoemulsion system. The modification in crystalline structure compared to bulk materials, as evidenced by the broad diffraction pattern, suggests potential improvements in dissolution rate and therapeutic efficacy of the encapsulated bioactive compounds^49,83^. The frequency distribution obtained from DLS analysis showed a narrow particle size distribution with the highest frequency occurring in the nanometer range. The frequency distribution demonstrates excellent size uniformity with minimal polydispersity, which is crucial for consistent therapeutic performance. The zeta potential measurements provided crucial information about the surface charge characteristics and colloidal stability of the nanoemulsion system. The measured zeta potential values indicated sufficient surface charge to maintain electrostatic repulsion between particles, preventing aggregation and ensuring long-term stability of the formulation. The negative surface charge observed is consistent with the presence of Spirulina extract components and contributes to the overall stability of the nanoemulsion through electrostatic stabilization mechanisms^84,85^. The height measurements correlated well with the particle size data obtained from DLS analysis, providing complementary information about the three-dimensional structure of the nanoparticles. The surface roughness analysis revealed minimal variation in particle surface topology, suggesting uniform distribution of the encapsulated compounds within the lipid matrix. The mechanical properties that can be assessed through AFM force measurements indicate appropriate particle rigidity for maintaining structural integrity while allowing controlled release of the bioactive compounds^86^. The comprehensive characterization study demonstrated successful synthesis of spirulina-coenzyme Q10 nanoemulsion with optimal physicochemical properties for pharmaceutical applications. The combination of morphological, structural, and colloidal characterization techniques provided a complete understanding of the nanoemulsion properties and confirmed the effectiveness of the sonochemical synthesis approach. The uniform particle size distribution, appropriate surface charge, and stable morphology collectively indicate the potential for enhanced bioavailability and therapeutic efficacy of the encapsulated bioactive compounds. The synergistic combination of spirulina extract and coenzyme Q10 within the nanoemulsion matrix offers potential advantages in terms of antioxidant activity and nutritional supplementation. The successful encapsulation of both compounds within a single delivery system demonstrates the versatility of the nanoemulsion platform for incorporating multiple bioactive ingredients while maintaining optimal physicochemical properties^87,88^.

In this investigation, heat stress adversely impacted the general health status of exposed Nile tilapia. This could be attributed to the adverse implications of high-water temperatures on oxygen solubility and availability, which in turn inhibit aerobic metabolism^89^. Earlier reports by Wiles, Bertram^90^, and Zafalon-Silva, Zebral^91^, confirmed that not only the physiological state and biochemical indices of farmed fish but also their ability to digest food are seriously hampered under chronic heat stress conditions. In the same context, Islam, Slater^92^ noted that thermal alteration significantly impaired various growth indexes (net weight, WG, and SGR) of European seabass (*Diecentrax labrax*). The survival rate and other body indexes, were also reduced, including hepatosomatic index, intestine somatic index, viscera somatic index, and spleen somatic index, .This might be explained by elevated metabolic activity brought on by a greater requirement for energy. In our work, this impact was mitigated by the addition of SCN, particularly SCN20 and SCN40. In light of this, feed additives and general immunostimulants such as algae that contain phycocyanin can be applied in aquaculture to mitigate temperature stress^93,94^. The enhanced growth rates upon feeding spirulina could be attributed not only to its high protein content (about 70%), vitamins, and mineral inclusion^95^ but also to its ability to stimulate lipase and amylase secretion^28,96^. Moreover, spirulina is also exceptionally digestible due to its smooth body and fragile cell wall^97^. Our results were supported by Okasha, Abdellatif^98^, who recorded the significant impact of *S. platensis on* Nile tilapia growth progress. As such, Velasquez, Chan^58^ recorded that, a lower level of spirulina (30%) in Nile tilapia’s diet significantly enhanced the growth performance, hepatosomatic and visceral-somatic indexes. A previous study in Great Sturgeon (*Huso huso*) by Adel, Yeganeh^27^ approved that, *S. platensis* at a dose of 100 g/kg fish diet, positively influences feed utilization and subsequently the growth performance rate. These outcomes were consistent with those reported by Teimouri, Amirkolaie^99^ who attributed the upregulated growth performance of rainbow trout to the dietary incorporation of *S. platensis*, which increased feed efficiency by promoting gut bacterial colonization. Furthermore, in line with these findings, dietary Coenzyme Q10 in Nile tilapia showed growth-promoting impacts^39,52^. The improved feeding frequency and activity via Coenzyme Q10, could be returned to several mechanisms; including promoting vitamin E regeneration^100,101^, enhancement of the plenty of microflora, inflammation reduction^102^, increasing activity of digestive enzymes^38^, as well as its involvement in the Krebs cycle, acquiring it a vital role in the metabolism of lipid, carbohydrate, and protein^48,103^. Interestingly, fish metabolic rate may be effectively improved by Co Q10, where this could be attributed to the fact that, one of the two essential components of CoQ10 is a benzoquinone ring, which transfers electrons in the mitochondrial membrane facilitating the respiratory chain. This promotes the synthesis of adenosine triphosphate (ATP), which contributes to the energy production of cells^104^. Moreover, Guo, Liang^52^, showed that the tiny intestinal mucus layer can be easily penetrated by small nanoparticles which are somewhat hydrophilic and have minimal negative charges. The right size, positive charge, and hydrophobic qualities are some of the variables that affect how well they pass through intestinal epithelial cells after passing through mucus. Accordingly, adding functional groups, stabilizers, or coatings with amphiphilic qualities may balance interactions with the mucus layer and epithelial cells enhancing nanoparticle delivery, and maximizing their efficacy in the gastrointestinal tract^53^. In our study, higher doses showed the highest improvement; suggesting that coenzyme Q10 could positively impact Nile tilapia with higher supplementation doses resulting in significant growth enhancement. This consequence aligns with El Basuini, Fattah^42^ unveiled that *L.ramada* dietary supplemented with 40 and 60 mg/kg Coenzyme Q10, showed upregulated levels of FBW, WG, and SGR after the experimental period (60 days). The growth-motivating characteristics of CoQ10 may be regarded to multiple processes, such as the regulation of microbiota variety, vitamin E resynthesis promotion, anti-inflammatory properties, and increased activity of digestive enzymes^38,45^. El Basuini, Teiba^60^ recorded the induction of Nile tilapia’s digestive enzyme activities after dietary supplementation with coenzyme Q10 at 20 to 40 mg/kg levels. These positive impacts of both spirulina and coenzyme Q10 in their nanoform could be accounted for by the enhanced capacity of nanominerals to take up substances that are frequently poorly uptaken in their natural or traditional states. Fish health can be improved and their immune systems strengthened by this increase in nutritional uptake^105^. Additionally, nanoparticles can enhance fish feed by promoting nutrient uptake into the circulation of fish and gut tissue absorption; consequently, lowering the amount of unabsorbed feed that is expelled through the digestive system^54^. Aquafeed minerals are also in nanoparticle form, which makes it easier for them to enter cells than their larger counterparts and speeds up absorption, perhaps enhancing fish performance and health^106^. Similarly, Abdel-Ghany, El-Sisy^107^, acknowledged that nano-curcumin performs significantly better than its free form in enhancing Nile tilapia survival ability under heat stress, inducing innate immunity, lowering stress indicators, and increasing growth efficiencies.

Owing to being an emerging low-cost dietary supplement, spirulina-CoQ10 combination has been used for increasing animal productivity, thus, contributing providing a sustainable and feasible food safety future. In this regard, our results showed that, in comparison to the SCN0 group, food supplementation with either SCN20 or SCN40 maintained an acceptable economic performance. SCN is thought to be a potential dietary supplement that was able to significantly increase the economic incomes of heat-stressed Nile tilapia, even if the profitability obtained from the SCN-fortified groups was marginally lower than that of the control diet (C25). These positive outcomes could be attributed to boosting fish growth, humeral, cellular, and local gut immunity (lymphocytes, goblet cells)^38,108,109^.

An increase in anaerobic free radical generation in fish exposed to high temperatures might produce an oxidative stress conditions and subsequent harmful effects as oxidative byproducts build up^110,111^. The aquatic animals’ antioxidant defense mechanisms can be activated to combat the excessive quantities of reactive oxygen species (ROS) produced during heat stress. This system depends on crucial enzymes, including superoxide dismutase, catalase, and glutathione peroxidase, as well as tiny reductive substances like glutathione and ascorbic acid^51^. Under thermal stress, ROS production surpasses the typical antioxidant capability; as a result, fish suffer oxidative stress-induced alterations^112^. Accordingly, fish activate their enzymatic antioxidant responses and radical-scavenging enzymes to ameliorate the harmful influences of ROS^55^. Therefore, the downregulation in their expressions might have resulted from heat stress, which was recovered in the SCN-supplemented group through upregulation of their expressions. Herein, heat-stressed fish displayed a significant reduction in SOD, CAT, and GST hepatic antioxidant contents with an increment in MDA level. However, the current findings suggested that, the inclusion of *S. platensis* in fish’s diets improved the anti-oxidative responses of fish. Herein, the strong antioxidant capabilities of spirulina, including the β-carotene component, phycocyanobilin, and phycocyanin, may be the cause of these encouraging results^113^. Glutathione peroxidase, superoxide dismutase, and catalase are valuable antioxidant indicators scavenging hydrogen peroxide and oxygen free radicals, which stop oxidative damage from occurring^114^. As a byproduct of the peroxidation process of lipids, malondialdehyde (MDA) has been employed as a biomarker in various biological samples to assess oxidative stress and the degree of tissue damage^115^. In the present investigation, the malondialdehyde level in Nile tilapia liver tissue at 32 °C was higher than that at 25 °C. This outcome agreed with Jinagool, Wipassa^11^ findings that Nile tilapia expressed a higher level of malondialdehyde in the blood at 38 °C compared with that between 24 °C and 26 °C. Additionally, *Luciobarbus capito* cultured at high ambient temperature showed higher values of MDA^116^. In line with our findings, striped catfish showed decreased antioxidative reactions (SOD, CAT, and GPx) with elevated MDA levels following heat shocks^117^. Additionally, Zhang, Li^118^ noted that once *Megalobrama amblycephala* was exposed to temperature stress, MDA levels were potentially increased together with decreased SOD and catalase activities. In contrast, the addition of SCN significantly reduced MDA levels. Similarly, grass carp expressed significant drops in serum levels of SOD and abrupt enhancement in glucose levels when heat stress was applied^119^. Wang, Liu^120^ recorded a crucial increase in the values of SOD and malondialdehyde (MDA) in heat-stressed rainbow trout. In a prior study, the records of GPX and SOD upregulated as dietary *S. platensis* in Nile tilapia increased, suggesting that 100 g kg^− 1^ of dietary *S. platensis* possesses antioxidant function^59^. Moreover, El Basuini, Fattah^42^ underscored that Coenzyme Q10 supplementation, especially at higher levels up to 80 mg/kg, effectively upregulated the antioxidant system, including SOD, CAT, and GPx in *L. ramada*. In agreement with our outcomes, an earlier study by Kumar, Kumar^121^, documented a remarkable improvement in the antioxidant potential of *Pangasianodon hypophthalmus* raised under temperature stress, which was indicated by augmenting the response of hepatic and branchial antioxidant genes following dietary Zn-NPs-supplementation.

Lysozyme is regarded as a crucial enzyme in innate immunity, possessing lytic and opsonic action against a variety of bacteria, in combination with phagocytosis and complement stimulation^122^. In addition, the breakdown of bacterial cell walls requires lysozyme activation^123^. Respiratory bursts (NBT), demonstrated to raise the rates of oxidation of phagocytes when activated by foreign chemicals, are frequently used in fish defensive systems^124^. Another indicator of a fish’s enhanced immune system is blood immunoglobulins (total Ig)^125^. Also, NO is a signaling molecule produced by immune cells with antimicrobial properties to combat pathogens^126^. As a main negative impact, fish subjected to a rise in temperature above their tolerance level suffered significant immune system suppression and the ensuing disease outbreaks^29,127^. Our outcomes revealed a sharp downregulation in NBT, Lysozyme, C3, NO, IgM, and PI records. The extensive formation of ROS during heat exposure is thought to be the cause of the suppressed immunological response in heat-stressed fish, which in turn impairs the immune system’s function, blood biochemistry, and physiological processes^117^. To prevent excessive inflammation, heat stress also causes the release of glucocorticoids, which decrease some immune response components. However, continuous exposure to heat stress can result in immunosuppression and an increased vulnerability to infections^129^. Similarly, Yang, Dong^130^, documented that heat stress not only reduced Largemouth bass’s immunological response to *Aeromonas veronii* but also lowered the coagulation, complement, and proinflammatory cytokines gene expression. Therefore, Largemouth bass mortality from infections is influenced by high water temperatures. Supporting our records, Elbahnaswy, Elshopakey^131^ reported that Nile tilapia’s phagocytic activity, respiratory burst, and total Ig levels were severely decreased following heat stress exposure. Comparably, heat stress reduced the phagocytic activity of Nile tilapia^132^ and striped catfish^117^. Furthermore, Nile tilapia exposed to 40 °C showed decreased IgM levels^107^. Similarly, Dominguez, Takemura^133^ listed that IgM levels increased when fish were reared at 18.4, 23, and 28 °C in contrast, when Nile tilapia was raised to 33 °C, IgM levels decreased, indicating that they had a suitable temperature range for producing immunological components. On the other hand, dietary SCN intervention, particularly with higher concentrations, counteracted this impact and highlighted a remarkably triggered immune response and consequently, improved fish health by increasing resistance to pathogens. The promising role of SCN as an immune stimulant and its ability to alleviate the effects of heat stress could stem from the inclusion of polyunsaturated fatty acids particularly γ-linolenic acid, and polysaccharides, in addition to valuable pigments such as carotenoids (β-carotene, zeaxanthin, and phycocyanin) and chlorophyll, which all can neutralize various free radicals either alone or in concert^134,135^. Beyond that, Liu, Liu^34^ recorded that *A. platensis* supplementation in yellow catfish (*Pelteobagrus fulvidraco*), especially at 40 g kg^− 1,^ increased plasma lysozyme (LZM) activity. Along with this, S. *platensis* has been recorded to promote lysozyme response in various aquatic species as Common carp^136^, white shrimp^137^, and great sturgeon^27^. These findings align with studies that underscored a significant upregulation not only in plasma activity of lysozyme, but also in the bactericidal and respiratory burst activities, with increased dietary levels of Coenzyme Q10 in *L. ramada*^42^, and in Nile tilapia^60^, which might be explained by dietary Coenzyme Q10’s immunomodulatory effects^138^. CoQ10 supplements have been shown to increase immunological parameters by preserving a healthy state of energy, lowering stress levels, improving mitochondrial respiration, shielding cell membranes, and boosting vitamin E regeneration levels^139^. Supporting our outcomes, Dube^140^ stated that, the immune-regulatory and antimicrobial forces of nanoparticles could improve fish immunity, hence, lower antibiotic usage, manage harmful bacteria, and finally improve health and lower the incidence of illness outbreaks.

On the level of tissue architecture, the histopathological findings revealed multiple lesions in the examined tissues up to chronic heat stress exposure for sixty days. Increased water temperature causes an overabundance of ROS, which could harm cellular components’ phospholipid membranes and result in metabolic and inflammatory dysfunctions in the tissues^141^. According to Nakano, Kameda^142^, cold-blooded fish species suffered significant harm from high temperatures, creating metabolic stress in the body. Fish that are exposed to HS regularly may develop pathological liver damage^143^. Similar outcomes with functional pathological variations in the fish liver and spleen were brought on by higher water temperatures in the rearing water^114,131^. Nonetheless, the aforementioned lesions were potentially ameliorated as a result of SCN dietary enrichment. In the same context, Awad, El-Mahallawy^144^ noted a normal histological structure of the liver of the spirulina-supplemented group except for mild signs of vacuolar degeneration in some hepatocytes after 8 weeks of the experiment. These records were confirmed by El Basuini, Shahin^43^, who clarified that the intestinal histological structure of European seabass was positively affected by dietary Coenzyme Q10 at 5 or 10 mg/kg. Similarly, a recent study by El Basuini, Fattah^42^ reported that the intestinal wall and mucosa of *L. ramada* remained normal and undamaged, and there was a corresponding rise in both villous height and intestinal villi branching as the amounts of CoQ10 supplementation in the fish diet rose (60 and 80 mg/kg). In addition, the same study also reported that upon inspection of the hepatic parenchyma, the liver seemed spongy, the hepatocytes around the central veins were still intact, and a notable progressive accumulation of glycogen was also evident with increasing CoQ10 supplementation levels. These results were in line withEl-Houseiny, Khalil^45^, and Huang, Ge^44^.

An earlier report by Adel, Sakhaie^145^, mentioned that essential nutrients and medicinal substances can be loaded and encapsulated efficiently thanks to these NPs’ great surface area-to-volume ratio. This feature helps to prevent deterioration and guarantees a controlled release of these components. Fish absorb nutrients and medicinal substances more readily when they are encapsulated, which also improves their stability. In aquaculture, this results in better growth performance, higher feed conversion ratios, and generally better health outcomes. Additionally, waste can be reduced by delivering these nutrients in a targeted manner. Regarding this aspect in our study, monitoring the fish post *S*. *agalactiae* challenge revealed a significant reduction in the rates of cumulative mortality in the SCN40 and SCN20 groups. In line with our results, previous research documented the possible immunostimulant effect of S. platensis inclusion in multiple fish species, increasing survivorship and improving protection against variant fish infections, including *E. tarda* in Nile tilapia^98^, *A. hydrophila* in Nile tilapia^146^, *A. hydrophila* in Gibel carp^142^, *Pseudomonas fluorescens*, *Aeromonas veronii* in Nile tilapia^96^, *Aeromonas sobria* in Common carp^136^, *Yersinia ruckeri* in rainbow trout^29^, and *Streptococcus iniae* in Great sturgeon^27^. Ragap, Khalil^147^ supported these records, claiming that small amounts of Spirulina provided a positive impact on overall health, as even small dietary doses (10 mg per fish per day) could bolster Tilapia’s defenses against infections. These findings may be attributable to the anti-inflammatory characteristics of Gamma-linolenic acid, phenolic compounds, and phycocyanin content of spirulina, which aid aquatic creatures in fending off infections^148,149^. In contrast, Kaushik and Chauhan^150^ reportedthat, although, *A. platensis* culture filtrate was efficient against *E. coli*, it was ineffective against *Klebsiella pneumoniae*,* Staphylococcus aureus*, and *Salmonella typhi* infections.

## Conclusions

Chronic exposure to high water temperatures is considered a crucial factor in tilapia aquaculture, where it highly affects the feed intake, immunity, and resilience to bacterial infections. Herein, dietary supplementation with spirulina-coenzyme Q10 nano emulsion at 20–40 mg/kg diet could enhance the growth rates and infection resistance in Nile tilapia via mitigating the diverse negative impacts of heat stress exposure. Consequently, net profitability will be increased and help in achieving aquaculture sustainability even in the face of adverse ecological conditions.

## Data Availability

The datasets generated or analyzed during the current study are not publicly available but are available from the corresponding author upon reasonable request.
